# Tomographic study of Jaw bone changes in patients with bisphosphonate-related osteonecrosis

**DOI:** 10.4317/jced.56265

**Published:** 2020-03-01

**Authors:** Guilherme Simpione, Rogério J. Caldas, Mariana Q. S. Soares, Izabel R. F. Rubira-Bullen, Paulo S. S. Santos

**Affiliations:** 1 Master student, Bauru School of Dentistry, University of Sao Paulo, Brazil; 2 PhD, Bauru School of Dentistry, University of Sao Paulo, Brazil; 3 Post-doctoral student Bauru School of Dentistry, University of Sao Paulo, Brazil; 4 Associate Professor, Department of Surgery, Stomatology, Radiology and Pathology, Faculty of Dentistry of Bauru – USP

## Abstract

**Background:**

Bisphosphonates (BP) are synthetic pyrophosphate-like substances with antiresorptive properties and specifically affect osteoclastic activity. In 2007, the American Association of Oral and Maxillofacial Surgeons (AAOMS) defined diagnostic criteria for Osteonecrosis of the Jaws Associated with Bisphosponates (BRONJ). BRONJ is mainly diagnosed by clinical features, but the detection of early bone changes by imaging may help prevent and better understand the disease. The objective of this study was to evaluate maxillary changes in CBCT in patients using BP.

**Material and Methods:**

All included patients were diagnosed with osteonecrosis and received bisphosphonate drugs in the last ten years. All imaging examinations were obtained by I-CAT and 3D Accuitomo. The multiplanar reconstructions were analyzed by an examiner without knowledge of the clinical aspects and location of the lesions.

**Results:**

The study sample consisted of 21 patients, the majority of the sample represented patients with cancer (76.2%), the other patients had osteoporosis (23.8%). Only four patients (19.04%) received alendronate, while intravenous bisphosphonates, such as zoledronate and pamidronate, represented the treatment of most of our sample. Most of our patients presented stage 1 and 2 MRONJ (85.7%), whose lesions were mainly observed in the mandible (52.4%). Fifty-seven percent of the patients had at least one bone change.

**Conclusions:**

In BRONJ, bone changes vary between exposed and non-exposed areas and one aspect of the study was: persistent extraction cavities in the BRONJ lesion region and high frequency of periodontal ligament space widening in areas that are not involved in BRONJ lesions. This reflects the very important role of dental and periodontal diseases in the pathophysiology of BRONJ. Thus, preventive measures should be prioritized for patients exposed to anti-resorptive drugs.

** Key words:**Cone-Beam computed tomography, osteonecrosis, bisphosphonate-associated osteonecrosis of the jaw.

## Introduction

Bisphosphonates (BP) are non-metabolic, synthetic, pyrophosphate-like substances with anti-resorptive properties. These drugs are indicated for several bone diseases such as: osteoporosis, Paget’s disease, hypercalcemia of malignant tumors, bone metastases and multiple myeloma(1,2). BP specifically affect osteoclastic activity, decreasing the number of osteoclasts by apoptosis induction. Consequently, the bone turnover is compromised. (3). In 2007, the American Association of Oral and Maxillofacial Surgeons (AAOMS) defined diagnostic criteria for BRONJ: current or previous BP treatment, exposed bone in the maxillofacial region that persisted for more than 8 weeks, and no history radiotherapy in the maxillary region (1). In 2014, AAOMS issued an updated paper on this topic (4). The intake of nitrogen-containing BP and the presence of local infection are known as the main risk factors for the disease. BRONJ is diagnosed primarily by clinical features, but radiological examinations are crucial to rule out hypotheses of other diseases as well as to evaluate the stage and extent of disease (5). The detection of early bone changes by means of radiographic imaging could help preventing and better understanding the disease. Therefore, the objective of this study is to evaluate changes in CBCT jawbones under the influence of bisphosphonates.

## Material and Methods

This study was approved by the Ethics Committee on Human Research of our institution (protocol number 1.959.558). All patients included were diagnosed with BRONJ in accordance with the AAOMS diagnostic and staging criteria (6). Only patients who received bisphosphonate drugs in the past ten years were included in this research. Data regarding clinical aspects of BRONJ, sex, age and medical history were obtained from patients’ charts. The imaging exams were obtained with i-CAT (Xoran Technologies, Ann Arbor, Mich., And Imaging Sciences International, Hatfield, PA) and 3D Accuitomo XYZ Slice View Tomograph (J. Morita, Kyoto, Japan). CT scans with field of views including the entire maxilla, or mandible or both were included for analysis. Multiplanar reconstructions were analyzed by one examiner unaware of the clinical aspects and location of the lesions. The presence of osteosclerosis, which is characterized by hyperdense images on CBCTs, laminae dura thickening without endodontics association, maxillary sinus cortical thickening, maxillary sinus alteration, bone sequestrum, bone resorption in the jaws, persistent extraction socket, and discontinuation of the maxillary sinus floor, cortical mandibular and cortical mandibular canal were identified and quantified separately for each quadrant. Each tomographic finding was evaluated for its presence in relation to the clinical location of the lesion: if it was present in the region (quadrant) of the lesion, in another quadrant, or present in both. The frequency of bone changes in each quadrant was quantified.

Stastistical analysis were performed by IBM SPSS Statístics 17 software for Windows. Chi-squared test was applied to verify the association between the bone changes and MRONJ stage, and the significance level was set at 5%.

## Results

The study sample consisted of 21 patients (Table 1). Of those, 23.8% correspond to men and 76.2% to women. Considering the diagnosis, most of the sample represented cancer patients (76.2%). The remaining patients suffered from osteoporosis (23.8%). Diabetes was also a comorbidity seen in five patients. Concerning risk factors for BRONJ, the use of corticosteroids was observed in 28.6% of the patients and seven patients (33.3%) had previous history of a recent dental extraction. A long-term bisphosphonate therapy (more than 3 years) was found in 57.1% of the patients. Only four patients (19.04%) received alendronate, while intravenous bisphosphonate such as zoledronate and pamidronate represented the treatment of the majority of our sample. Most of our patients presented with stage 1 and 2 BRONJ (85.7%), whose lesions were mainly seen in the mandible (52.4%). Dental implants as well as remaining tooth root were uncommonly seen in the area of BRONJ lesions (4.8%).

**Table 1:**
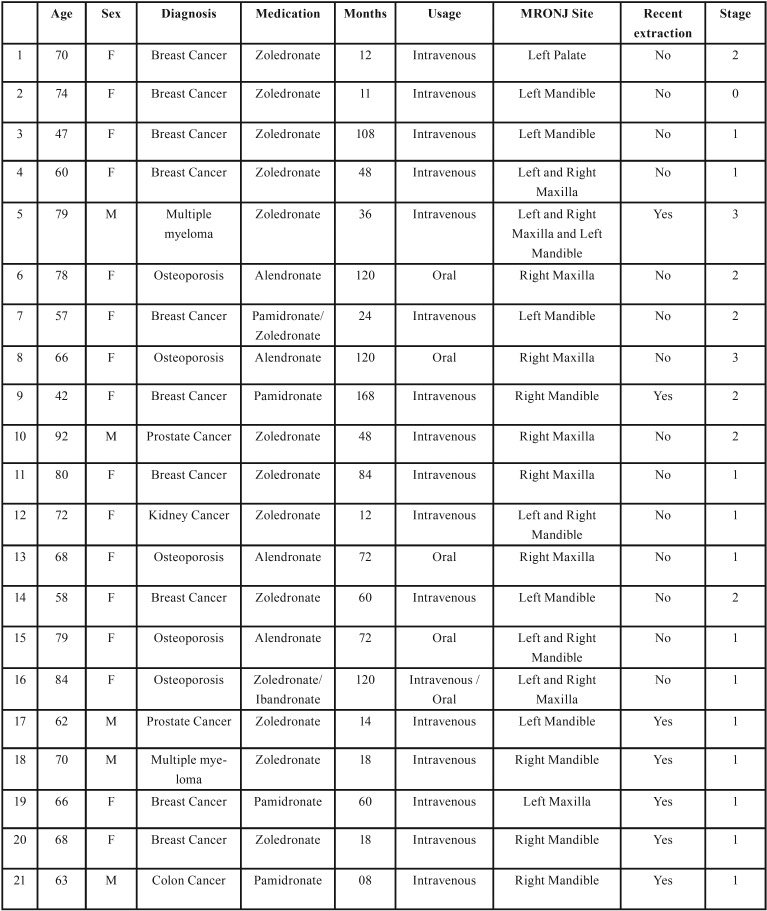
Clinical and prospective data from 21 patients.

As bone changes, all of the following could be observed: widening of the periodontal ligament space without endodontics association, thickened laminae dura, maxillary sinus cortical thickening, maxillary sinus alteration, thickening of the mandibular cortical, thickening of the mandibular canal cortical, osteosclerosis, bone sequestrum, bone resorption, persistent extraction socket, and discontinuation of the maxillary sinus floor (Table 2). In 57% of the patients one or more bone alterations were present in the lesion region and in another region. Differences among the quadrant of BRONJ lesions and the others could be detected. In the region of BRONJ lesions, the most frequent bone changes were bone sequestrum (52.4%), persistent extraction socket (42.9%) and osteosclerosis (28.6%). On the other hand, changes such as widening of periodontal ligament space (33.3%) and maxillary sinus alteration (23.8%) followed by thickened mandibular canal cortical (19%) and bone resorption (19%) were the most recurrent in the other quadrants. Concerning the less common bone changes, a thickened lamina dura and maxillary sinus cortical were characteristics of quadrants with and without BRONJ lesion. While bone resorption could be barely seen near BRONJ lesions, bone sequestrum could be rarely noted in non-affected quadrants.

**Table 2:**
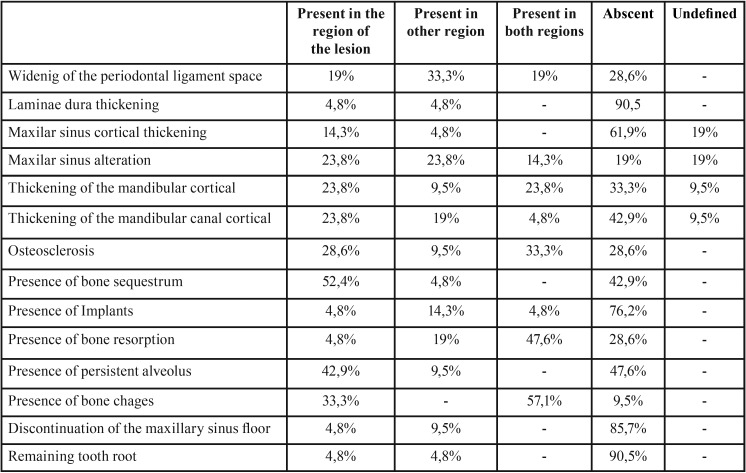
Prevalence of jaw changes on cone beam computed tomography.

## Discussion

This work identified jaw bone changes among patients with BRONJ, describing tomographic findings in regions with BRONJ lesions as well as in the non-affected ones. These areas without stablished BRONJ lesion might reveal changes correspondent to effects of the antiresorptive drugs on bone. Comparing both regions, it was expected to find out alterations in bone that could point out early signs of BRONJ. In fact, this study showed tomographic signs near BRONJ lesions that were absent in non-affected sites and vice versa. Interestingly, Guo *et al.* could not detect any changes in the bony architecture on the non-osteonecrosis sites in the same patients with BRONJ by CBCT.

Even though studies on imaging of BRONJ have been conducted, none have contributed to determine early diagnosis of this condition (7). Their conclusions are even contradictory. Some researches postulated that the first evidence of BRONJ would be osteosclerosis and an active bone resorption would be present at advanced stages (8–12). Others claimed that the degree of sclerosis might increase as clinical severity of the condition progressed (13–16). In the present study, osteosclerosis was very frequent in BRONJ quadrants but not in non-compromised sites, so that it seemed to be related to advanced BRONJ stages. Nonetheless, Wilde *et al.* presumed that this sign is generally insignificant in indicating the severity and extent of the BRONJ lesion, since it was irregularly distributed across all BRONJ stages in their study. Actually, the presence of osteosclerosis in clinically symptomatic areas of the jaws has been described as a consistent tomographic finding, both in initial and advanced forms of osteonecrosis (14,17).

Besides sclerosis, osteolysis and sequestrum formation were frequent findings in stage 1 and 2 BRONJ, and universal in Stage 3 BRONJ (11,17). Corroborating these outcomes, our work showed that bone sequestrum was markedly a common finding near BRONJ lesions and the less recurrent one in other regions, but bone resorption could be frequently noted in all areas. However, these well-recognized tomographic features have not been found consistently and have been mainly associated with advanced stages of this disease (12,14).

Also, thickening of the lamina dura and alveolar crest have been considered as the most common radiographic features in patients taking bisphosphonate (12,18). They might indicate a higher risk for BRONJ and have been related to its early stages (9,10,12,19). Differently, a thickened lamina dura was hardly found near MRONJ lesions and in non-affected sites in our study. However, we could often see a widening of periodontal ligament space in areas other than involved in BRONJ lesions. Associated with the high frequency of persistent extraction sockets (42.9%) in the region of BRONJ lesions also observed in our study, a main role of dental and periodontal diseases in the development of BRONJ could be unveiled for our sample. This is in line with recent outcomes evidenced by a systematic review (7).

A significant increase in mandibular cortical thickness was verified by CBCT in case-control studies (16,20,21). Confirming these data, two retrospective studies assessed the mandibular cortical width by comparing CBCTs obtained from patients with BRONJ, patients under antiresorptive therapy without symptoms of BRONJ and patients without history of antiresorptive medication use and symptoms of BRONJ (22,23). Their results pointed out a significant difference in cortical bone width between BRONJ and control groups, but no correlation could be found between non-BRONJ and control categories. Additionally, the low interobserver reliability between the two observers is a major bias in one of these studies. Another case-control study (24) evaluating the mandibular cortical width and the height from the inferior mandibular border to the mental foramen in 46 Caucasian women receiving oral bisphosphonates without BRONJ demonstrated that both measurements should not be used to predict the risk of this condition. Our study revealed a thickened mandibular cortical bone near BRONJ lesions and even in other areas. In other words, mandibular cortical thickness is a measurement that hardly differentiate patients taking antiresorptive agents without BRONJ from other groups. Consequently, it is challenging to predict the incidence of BRONJ using only this parameter.

A thickened mandibular canal cortical has been advocated as an early sign of BRONJ (7,12). Nonetheless, it was a recurrent sign in areas with and without osteonecrosis in the present study. A case-control study measured the diameter and width of mental foramen as well as the diameter of incisive canal, and just the narrowing of incisive canal was observed in patients with BRONJ (21). This sign was deemed late as a result of progressive sclerotic changes (9,25).

The formation of new periosteal bone could not be found in the present study. As it has been detected in advanced BRONJ stage (9,12,14,15), this might reflect the only two patients at BRONJ stage 3. The involvement of maxilla in our study manifested mainly as maxillary sinus alteration which was a common finding in areas with and without osteonecrosis. The thickening of maxillary sinus cortical was more recurrent in BRONJ areas. These maxillary manifestations were characteristics of middle to advanced BRONJ stages (9,12,14).

Finally, we acknowledge certain limitations to this study. First, we must admit that this study reveals the disadvantages of retrospective studies only with tomographic analysis without clinical features, and second, we must address the problems of single-center BRONJ studies in general. Additionally, we consecutively included 16 female patients and only 5 male patients with BRONJ in our study, leading to a marked female majority in our patient population. Although jaw bones of both genders might be considered as equally susceptible to side effects from bisphosphonate treatment, subtle gender-specific differences could not be ruled out with certainty.

In BRONJ, bone alterations vary greatly in exposed and unexposed in intraoral areas and most of them can be seen throughout all stages of the disease. However, one aspect of the present study should call our attention: the combination of high frequency of persistent extraction sockets in the region of BRONJ lesions and high frequency of widening of periodontal ligament space in areas other than involved in BRONJ lesions. This reflects the very important role in preventing dental and periodontal diseases (dental and periodontal tissues) in patients with or at risk of developing BRONJ. Thus, preventive measures should be prioritized for patients exposed to anti-resorptive drugs.
